# Associations between bullying, depressive symptoms, parental supervision, and suicidal behaviors in Chinese adolescents: a cross-sectional study

**DOI:** 10.3389/fpsyg.2026.1984417

**Published:** 2026-10-29

**Authors:** La Mang, Ru Zheng, Jiali Duan, Xin Guo, Yi Xing

**Affiliations:** 1 Department of Child and Adolescent Health, School of Public Health, Peking University Health Science Center, Beijing, China; 2 Beijing Center for Disease Prevention and Control, Beijing, China; 3 Chinese Center for Disease Control and Prevention, Beijing, China

**Keywords:** bullying, Chinese adolescents, depressive symptoms, parental supervision, suicidal behaviors

## Abstract

**Background:**

School bullying is associated with elevated risk of adolescent mental health problems, yet the complex interrelationships between bullying experiences, depressive symptoms, and parental factors in the Chinese cultural context require further investigation. This cross-sectional study examined associations between traditional and cyberbullying (as victim or perpetrator), depressive symptoms, parental supervision, and suicidal behaviors among Chinese adolescents.

**Methods:**

Data from 12,019 students (Grades 5–12) recruited from 16 administrative districts in Beijing through a cross-sectional survey using multi-stage cluster random sampling were included in the final analyses. Participants completed standardized measures of traditional and cyberbullying (victimization and perpetration), depressive symptoms, suicidal behaviors, and parental supervision. Associations among variables were examined using chi-square tests, ANOVA, and correlation analyses. Indirect and conditional associations were examined using PROCESS Models 4 and 59, respectively, with 5,000 percentile bootstrap resamples, adjusting for demographic and psychosocial covariates.

**Results:**

Among 12,019 participants, 1,322 (11.0%) reported suicidal behaviors. All forms of bullying were significantly associated with both depressive symptoms and suicidal behaviors. Depressive symptoms were associated with both bullying experiences and suicidality, consistent with a potential mediating pathway. The associations between bullying involvement and depressive symptoms, as well as between depressive symptoms and suicidal behaviors, were stronger at higher levels of perceived parental supervision.

**Conclusion:**

This cross-sectional study documented significant associations between bullying, depressive symptoms, parental supervision, and suicidal behaviors among Chinese adolescents. While depressive symptoms may represent a key psychological pathway linking bullying to suicidality, the conditional associations observed suggest that parental supervision may interact with these processes in complex ways. Longitudinal research is needed to clarify causal pathways and inform culturally sensitive prevention strategies.

## Background

Bullying is a repetitive and intentional act of aggression involving an imbalance of power between the perpetrator and the victim (Olweus, 2010). It manifests primarily in two forms: traditional bullying, characterized by physical, verbal, or relational aggression occurring face-to-face, and cyberbullying, which occurs via digital platforms such as social media, messaging apps, and online forums. Both forms exert significant negative effects on adolescent well-being, contributing to physical injury, emotional distress, and social dysfunction. Prior research consistently links bullying involvement to elevated risks of depressive symptoms and suicidal behaviors, including ideation, planning, and attempts (Han et al., 2025; Holt et al., 2015). Globally, a cross-national study involving 317,869 adolescents from 83 countries reported a pooled prevalence of 30.5% for bullying victimization in the preceding 30 days (Biswas et al., 2020). A meta-analysis of school-aged populations in Chinese communities reported pooled prevalence estimates of 20.8% for traditional bullying victimization and 9.6% for cyberbullying victimization, compared with 10.3 and 8.4% for the corresponding perpetration categories (Xing et al., 2023). In addition, a recent meta-analysis estimated that 26.17% of Chinese children and adolescents experienced depressive symptoms (Zhou et al., 2024). These estimates varied substantially across studies, highlighting differences in populations and measurement approaches.

Although previous studies have documented the prevalence and mental health correlates of bullying among Chinese adolescents, less is known about whether the associations of traditional and cyberbullying victimization and perpetration with depressive symptoms and suicidal behaviors vary according to perceived parental supervision. However, existing studies differ in their populations and measurement approaches, which may contribute to variations in reported bullying prevalence and limit direct comparisons across studies. Moreover, previous research has examined diverse parental factors, and findings concerning parental support or control may not necessarily apply to adolescents’ perceived parental knowledge. To address this gap, the present study examines four bullying subtypes separately—traditional victimization, cyber victimization, traditional perpetration, and cyber perpetration—among Chinese students in Grades 5–12.

Parental supervision refers to the degree to which parents monitor and stay informed about their children’s activities, whereabouts, and peer relationships. Within the framework of the social ecological diathesis–stress model (Swearer and Hymel, 2015), such supervision is commonly viewed as a protective factor that may buffer the psychological impact of bullying. However, emerging research highlights that parental monitoring and broader parenting practices may have complex associations with adolescents’ emotional and psychological outcomes (Yap et al., 2014; Teuber et al., 2022). Rather than offering uniform protection, the nature and context of supervision may shape how youth respond to interpersonal stressors like bullying—potentially influencing pathways toward depression or suicidal behaviors. These nuances underscore the need for deeper investigation into how parent–child dynamics are related to adolescents’ vulnerability or resilience in the face of peer aggression.

Accordingly, this study aims to advance understanding of how different bullying roles—both victim and perpetrator, across traditional and cyber contexts—are associated with adolescent mental health. It further explores whether the associations between bullying exposure, depressive symptoms, and suicidal behaviors vary according to levels of parental supervision. Examining these conditional associations can inform culturally sensitive prevention and intervention strategies that promote youth well-being in Chinese educational contexts. Given the cross-sectional nature of this study, findings should be interpreted as documenting patterns of association rather than establishing causal relationships.

## Methods

### Study design and setting

This study employed a cross-sectional design. Data were collected through a large-scale school-based survey in Beijing, China, during the 2017–2018 academic year. Participants completed paper-based questionnaires independently during scheduled class periods under the supervision of trained public health staff from the Beijing and District Centers for Disease Control and Prevention (CDCs).

Prior to participation, students were clearly informed via written questionnaire instructions that their involvement was voluntary, that all responses would be kept confidential, and that they could opt out at any time without consequences. Verbal informed consent was obtained from both parents and students. No personal identifying information was collected to protect privacy and confidentiality.

All procedures were reviewed and approved by the Institutional Review Board of Peking University (IRB00001052-17011) and were conducted in accordance with the Declaration of Helsinki.

### Participants

Participants were recruited from general primary and secondary schools (Grades 5 to 12) and vocational secondary schools (Grades 10 to 12) across 16 administrative districts in Beijing using a multi-stage cluster random sampling method. According to the original sampling protocol, two primary schools, two junior secondary schools, and two senior secondary schools were randomly selected from each of Beijing’s 16 administrative districts. In the six central urban districts, general and model schools were considered in the sampling process, whereas in the 10 suburban districts, schools from urban and township areas were considered. Vocational secondary schools were sampled separately from six districts, with approximately 60 students per grade level.

Within each selected school, one or two classes per grade were randomly selected, and all students in the selected classes were invited to participate. The sample size was calculated using the single-proportion formula, assuming an expected proportion of 43.2% based on previous research on school bullying among Chinese adolescents (Qiao et al., 2009), a 95% confidence level, and a margin of error of 5%, yielding a minimum sample size of 378. After accounting for a design effect of 2 and an anticipated response rate of 90%, the required sample size was increased to 840 per stratum. Considering 12 strata defined by geographic location (urban/suburban), sex, and educational stage, the planned sample size for general schools was 10,080. An additional 1,080 students were planned to be recruited from six vocational schools, resulting in a total target sample size of 11,160. The available dataset contained 13,514 participant records. After excluding 1,495 records (11.1%) with missing data on variables required for the analyses, 12,019 students (88.9%) were included in the final analytical sample (mean age = 14.2 years, SD = 1.8; 53.6% female).

### Measures

#### Traditional bullying behaviors

Traditional bullying was assessed using the Traditional Bullying Perpetration and Victimization Scales adapted from the Olweus Bully/Victim Questionnaire (OBVQ) (Cikili-Uytun et al., 2022), a widely validated instrument for measuring bullying behaviors among school-aged children. Each scale included 9 items evaluating the frequency of traditional bullying behaviors over the past 6 months, such as physical aggression, verbal teasing, and social exclusion. Items were rated on a 3-point Likert scale (1 = never, 2 = sometimes, 3 = often). Total scale scores were treated as continuous variables to reflect varying levels of bullying involvement. Higher scores indicated greater frequency of perpetration or victimization. The Cronbach’s alpha was 0.82 for perpetration and 0.79 for victimization.

#### Cyberbullying behavior

Cyberbullying was measured using adapted versions of scales developed by Fahy et al. (2016) and Mishna et al. (2012). Each scale contained 7 items assessing the frequency of cyberbullying perpetration and victimization over the previous 6 months, covering behaviors such as online insults, rumor-spreading, and digital exclusion. Responses were rated on a 3-point Likert scale. Scores were treated as continuous indicators, with higher totals reflecting increased frequency of cyberbullying involvement. The internal consistency reliability was 0.89 for perpetration and 0.81 for victimization.

#### Depressive symptoms

Depressive symptoms were assessed using the Center for Epidemiologic Studies Depression Scale (Radloff, 1977), which measures the frequency of depressive experiences over the past week. The scale consists of 20 items, each rated on a 4-point Likert scale (0 = less than 1 day, 1 = 1–2 days, 2 = 3–4 days, 3 = 5–7 days). Four items (4, 8, 12, and 16) were reverse-coded. Total scores range from 0 to 60, with higher scores indicating greater severity of depressive symptoms. In this study, CES-D scores were analyzed as continuous variables. The Cronbach’s alpha was 0.82.

#### Suicidal behaviors

Suicidal behaviors were assessed using three binary-response items: (1) “Have you seriously considered suicide in the past 12 months?” (2) “Have you made a plan for suicide in the past 12 months?” and (3) “Have you attempted suicide in the past 12 months?” Participants who responded “yes” to any item were classified as having reported at least one suicide-related outcome.

#### Parental supervision

Parental supervision was assessed using a 2-item scale adapted from the scale developed by Stattin and Kerr (2000), which captured adolescents’ perceptions of their parents’ knowledge of their whereabouts and friendships. The two items were: (1) “After school or on weekends, my parents don’t ask where I go or when I’ll return,” and (2) “My parents know nothing about my close friends.” Each item was rated on a 5-point Likert scale (1 = strongly agree to 5 = strongly disagree). Scores were summed, with higher scores reflecting greater parental supervision. In this study, Cronbach’s alpha for the two-item scale was 0.697. Given its brevity and reliance on adolescent self-reports, this measure should be interpreted as an indicator of perceived parental knowledge and supervision rather than a comprehensive assessment of parental monitoring or parenting practices.

#### Covariates

The following variables were controlled for in the analysis: gender (female, male), school grade (grade 5–6, 7–9, and 10–12), single-child status (yes, no), self-efficacy, mother’s educational level (middle school or below, high school, university or above, unsure), father’s educational level (same categories), family structure (nuclear family, extended family, single-parent/restructured family, other), school type (general school, key school, vocational secondary school), residence status (boarding or non-boarding), number of close friends (none to 5 or more).

### Statistical analysis

All statistical analyses were conducted using SPSS version 26.0. Descriptive analyses were used to summarize participant demographics, bullying involvement, and psychological outcomes. Frequencies and percentages were reported for categorical variables; means and standard deviations were used for continuous variables.

To examine bivariate associations between predictor variables and the binary outcome variable (suicidal behaviors), Pearson chi-square tests were used for categorical predictors (e.g., gender, grade, only-child status, family structure, number of close friends, school type, residence status). For continuous predictors (e.g., bullying involvement, depressive symptoms, self-efficacy, parental supervision), one-way analysis of variance (ANOVA) was conducted to compare mean levels between students with and without suicidal behaviors. Pearson correlation analyses were also performed to evaluate linear relationships among continuous study variables.

To examine associations among bullying, depressive symptoms, parental supervision, and suicidal behaviors while adjusting for covariates, logistic regression analyses were conducted using the PROCESS macro version 4.1 for SPSS (Hayes, 2015). Given that the outcome variable (suicidal behaviors) was binary, logistic regression was used for all models predicting suicidal behaviors; regression coefficients for paths predicting the binary outcome are therefore expressed in log-odds units.

PROCESS Model 4 was used to examine whether depressive symptoms were associated with both bullying experiences and suicidal behaviors in a pattern consistent with statistical mediation. Separate models were estimated for each of the four bullying variables (traditional victimization, cyber victimization, traditional perpetration, cyber perpetration) as the independent variable, with depressive symptoms as the mediator and suicidal behaviors as the outcome.

To explore whether associations varied according to levels of parental supervision, PROCESS Model 59 was used to test conditional associations (moderated mediation models; see Figure 1). In these models, parental supervision (measured continuously) was specified as a moderator of the associations between (a) bullying and depressive symptoms, (b) depressive symptoms and suicidal behaviors, and (c) bullying and suicidal behaviors (direct path). Conditional direct and indirect associations were estimated at three levels of parental supervision: low (16th percentile), medium (50th percentile), and high (84th percentile).

**Figure 1:**
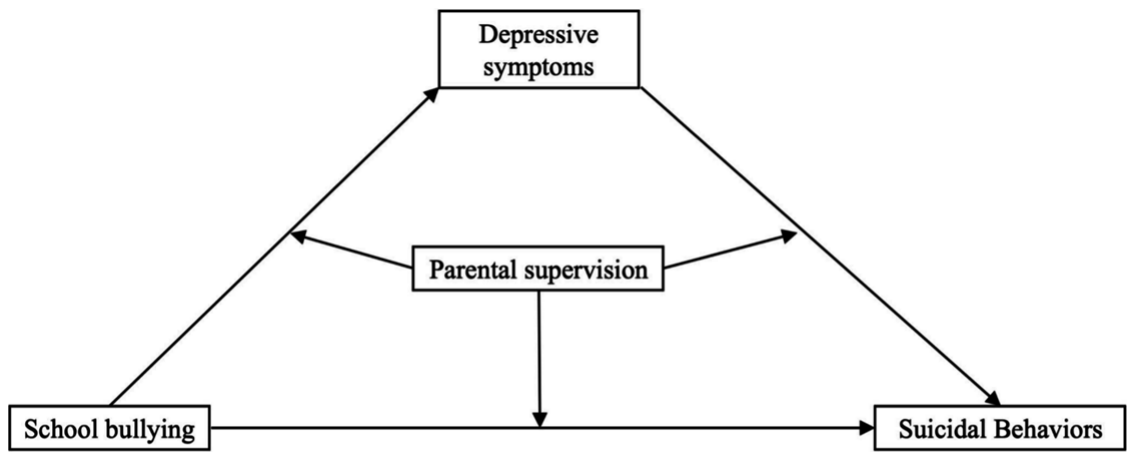
Hypothesized conceptual model of moderated mediation. The model illustrates the associations between bullying involvement and suicidal behaviors, both directly and indirectly through depressive symptoms. Parental supervision was specified as a moderator of three associations: (a) bullying involvement and depressive symptoms, (b) depressive symptoms and suicidal behaviors, and (c) bullying involvement and suicidal behaviors. Separate PROCESS Model 59 analyses were conducted for traditional victimization, cyber victimization, traditional perpetration, and cyber perpetration.

Prior to conducting correlation and PROCESS regression analyses, all continuous predictors—including the four bullying indices, depressive symptoms, self-efficacy, and parental supervision—were standardized using z-transformation to facilitate interpretation and comparison of coefficients across models.

Indirect associations were estimated using percentile bootstrap 95% confidence intervals based on 5,000 resamples. All statistical tests were two-sided, with *p* < 0.05 considered statistically significant. Indirect associations were considered statistically significant when their 95% bootstrap confidence intervals did not include zero. Categorical covariates with more than two categories were dummy-coded, using Grades 5–6 for educational stage, nuclear family for family structure, general school for school type, and middle school education or below for parental educational attainment as the reference categories. Paternal and maternal educational attainment were coded separately. Binary covariates were entered as indicator variables. The number of close friends was treated as a numerical covariate in the PROCESS analyses and as a categorical covariate in the supplementary multilevel logistic models. To account for the hierarchical structure of students nested within schools, supplementary multilevel analyses were conducted as sensitivity analyses. Two-level random-intercept models were fitted, with students at Level 1 and schools at Level 2. Linear mixed-effects models were used for depressive symptoms, whereas generalized linear mixed-effects models with a binomial distribution and logit link were used for suicidal behaviors. School identifiers were derived from recorded school names. These models adjusted for the study covariates and included the corresponding interaction terms to assess the robustness of the main findings. An unconditional linear mixed-effects model was also fitted to estimate the intraclass correlation coefficient (ICC) for depressive symptoms. Because class-level identifiers were unavailable, additional clustering within classes could not be explicitly modeled.

## Results

### Characteristics of the participants

The final analytical sample comprised 12,019 students. Among them, 6,447 were female students (53.6%), and 8,225 students (68.5%) reported being an only child. Regarding educational stages, 2,667 students (22.2%) were in grades 5–6, 4,121 (34.3%) were in middle school (grades 7–9), and 5,231 (43.5%) were in high school (grades 10–12). Most participants (9,525 students, 79.3%) did not reside in school. With respect to school type, 5,682 students (47.3%) attended general schools, 5,403 (45.0%) were enrolled in model schools, and 934 (7.8%) attended vocational secondary schools. Table 1 shows the demographic characteristics of the participants in this study.

**Table 1:** Characteristics of study participants and associations with suicide-related outcomes (*N* = 12,019).

Variable	*n* (%) or M (SD)	With SB	Without SB	χ^2^ or F	*p*
Categorical variables					
Gender				21.265	<0.001
Male	5,572 (46.4%)	534 (9.6%)	5,038 (90.4%)		
Female	6,447 (53.6%)	788 (12.2%)	5,659 (87.8%)		
Grade				63.246	<0.001
Grades 5–6	2,667 (22.2%)	182 (6.8%)	2,485 (93.2%)		
Grades 7–9	4,121 (34.3%)	480 (11.6%)	3,641 (88.4%)		
Grades 10–12	5,231 (43.5%)	660 (12.6%)	4,571 (87.4%)		
Only-child status				7.170	0.007
Non-only-child	3,794 (31.6%)	460 (12.1%)	3,334 (87.9%)		
Only-child	8,225 (68.5%)	862 (10.5%)	7,363 (89.5%)		
Family structure				67.186	<0.001
Nuclear family	6,719 (55.9%)	692 (10.3%)	6,027 (89.7%)		
Extended family	3,603 (30.0%)	352 (9.8%)	3,251 (90.2%)		
Single-parent/Reconstituted	578 (4.8%)	112 (19.4%)	466 (80.6%)		
Other	1,119 (9.3%)	166 (14.8%)	953 (85.2%)		
School type				13.779	0.001
General school	5,682 (47.3%)	571 (10.0%)	5,111 (90.0%)		
Model school	5,403 (45.0%)	623 (11.5%)	4,780 (88.5%)		
Vocational school	934 (7.8%)	128 (13.7%)	806 (86.3%)		
Boarding (resides at school)				31.186	<0.001
No	9,525 (79.3%)	970 (10.2%)	8,555 (89.8%)		
Yes	2,494 (20.8%)	352 (14.1%)	2,142 (85.9%)		
Number of close friends				194.383	<0.001
0	613 (5.1%)	155 (25.3%)	458 (74.7%)		
1	932 (7.8%)	146 (15.7%)	786 (84.3%)		
2	1,798 (15.0%)	225 (12.5%)	1,573 (87.5%)		
3	2,152 (17.9%)	236 (11.0%)	1,916 (89.0%)		
4	1,066 (8.9%)	107 (10.0%)	959 (90.0%)		
≥5	5,458 (45.4%)	453 (8.3%)	5,005 (91.7%)		
Father’s education				8.733	0.033
Middle school or below	3,061 (25.5%)	333 (10.9%)	2,728 (89.1%)		
High school	3,237 (26.9%)	397 (12.3%)	2,840 (87.7%)		
University or above	4,487 (37.3%)	455 (10.1%)	4,032 (89.9%)		
Not sure	1,234 (10.3%)	137 (11.1%)	1,097 (88.9%)		
Mother’s education				2.324	0.508
Middle school or below	3,170 (26.4%)	366 (11.5%)	2,804 (88.5%)		
High school	3,130 (26.0%)	345 (11.0%)	2,785 (89.0%)		
University or above	4,526 (37.7%)	492 (10.9%)	4,034 (89.1%)		
Not sure	1,193 (9.9%)	119 (10.0%)	1,074 (90.0%)		
Continuous variables					
Traditional victimization	10.30 (2.15)	11.71	10.13	674.971	<0.001
Cyber victimization	7.48 (1.35)	8.30	7.38	572.338	<0.001
Traditional perpetration	9.43 (1.37)	10.00	9.36	257.699	<0.001
Cyber perpetration	7.14 (0.90)	7.44	7.10	168.051	<0.001
Depressive symptoms	11.41 (9.20)	21.12	10.21	1923.248	<0.001
Self-efficacy	26.75 (7.36)	24.74	27.00	112.149	<0.001
Parental supervision	8.72 (1.74)	8.10	8.79	188.244	<0.001

For categorical variables, the Total column presents *n* (% of the full sample), whereas the outcome columns present *n* (% within each demographic category). For continuous variables, the Total column presents M (SD), and the outcome columns present group-specific means. Suicide-related outcomes include suicidal ideation, suicide planning, or suicide attempts.

### Correlations between variables and outcome (suicidal behaviors)

In this study, 1,322 students (11.0%) reported having suicidal behaviors. Pearson chi-square tests revealed that gender, grade, only-child status, family structure, school type, boarding status, and number of close friends were significantly associated with suicidal behaviors (all *p* < 0.01). For example, female students were more likely to report suicidal behaviors than male students (12.2% vs. 9.6%, χ^2^ = 21.27, *p* < 0.001). Students in higher grades also showed greater prevalence, with the highest proportion observed in high school (12.6%, χ^2^ = 63.25, *p* < 0.001). Students from single-parent or reconstituted families (19.4%) and those from other non-traditional family structures (14.8%) were more likely to report suicidal behaviors compared to their peers from nuclear (10.3%) or extended families (9.8%). Additionally, students with fewer close friends exhibited significantly higher rates of suicidal behaviors (χ^2^ = 194.38, *p* < 0.001), with the highest prevalence observed among those reporting no close friends (25.3%). Father’s education level showed a statistically significant association (*p* = 0.033), whereas mother’s education level did not (*p* = 0.508). Students who resided in school (boarding students) also showed higher rates of suicidal behaviors compared to non-boarding students (14.1% vs. 10.2%, χ^2^ = 31.19, *p* < 0.001). Table 1 summarizes these associations.

For continuous variables, one-way analysis of variance (ANOVA) revealed significant differences between students with and without suicidal behaviors across all study variables (all *p* < 0.001). Students reporting suicidal behaviors had significantly higher scores on all four types of bullying involvement (traditional victimization: M = 11.71 vs. 10.13, *F* = 674.97, *p* < 0.001; cyber victimization: M = 8.30 vs. 7.38, *F* = 572.34, *p* < 0.001; traditional perpetration: M = 10.00 vs. 9.36, *F* = 257.70, *p* < 0.001; cyber perpetration: M = 7.44 vs. 7.10, *F* = 168.05, *p* < 0.001). Additionally, students with suicidal behaviors reported markedly elevated depressive symptoms (M = 21.12 vs. 10.21, *F* = 1923.25, *p* < 0.001), lower self-efficacy (M = 24.74 vs. 27.00, *F* = 112.15, *p* < 0.001), and lower parental supervision (M = 8.10 vs. 8.79, *F* = 188.24, *p* < 0.001). These findings are presented in Table 1.

In addition, Pearson correlations among the continuous study variables and their correlations with the binary suicide-related outcome are presented in Supplementary Table 1.

### Tests of mediation model

Figure 2 presents the results of four mediation analyses examining the role of depressive symptoms in the relationship between traditional bullying and cyberbullying (both for victimization and perpetration) and suicidal behaviors.

**Figure 2:**
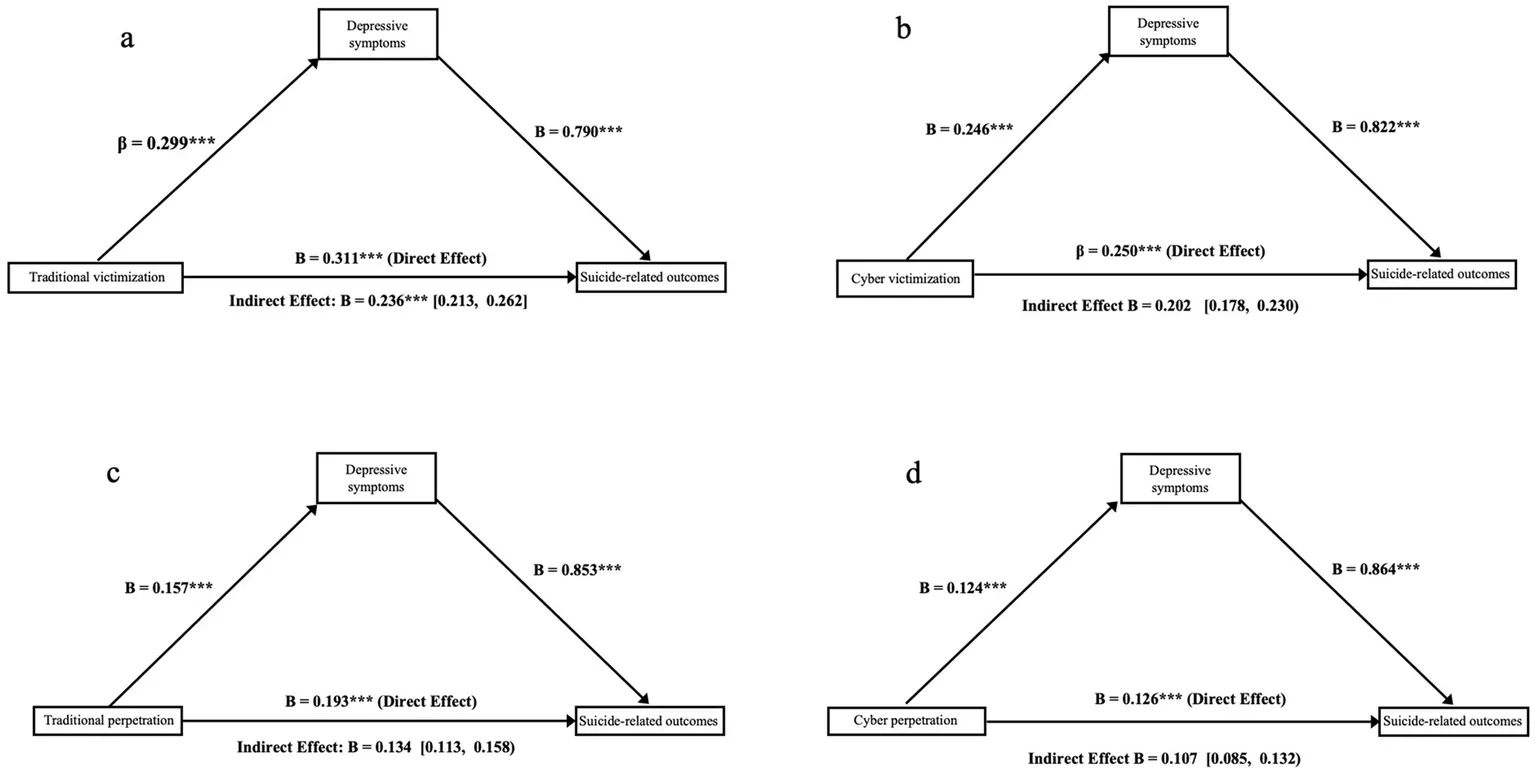
Path coefficients from mediation models (PROCESS Model 4): **(a)** Traditional bullying victimization, **(b)** cyberbullying victimization, **(c)** traditional bullying perpetration, and **(d)** cyberbullying perpetration. Continuous predictors were standardized before analysis. Regression coefficients (B) are reported, with coefficients predicting suicide-related outcomes expressed in log-odds units. Indirect associations are presented with 95% percentile bootstrap confidence intervals based on 5,000 resamples. All models adjusted for demographic and psychosocial covariates. ***p < 0.001.

As shown in Figure 2a, traditional bullying victimization was positively associated with depressive symptoms (B = 0.299, *p* < 0.001), which in turn was positively associated with suicidal behaviors (B = 0.790, *p* < 0.001). The direct effect of traditional bullying victimization on suicidal behaviors was also significant (B = 0.311, *p* < 0.001), consistent with a statistical indirect association. The indirect effect via depressive symptoms was significant (indirect effect B = 0.236, 95% CI [0.213, 0.262]).

Figure 2b shows that cyberbullying victimization was positively associated with depressive symptoms (B = 0.246, *p* < 0.001), which was also associated with suicidal behaviors (B = 0.822, *p* < 0.001). The direct effect of cyberbullying victimization remained significant (B = 0.250, *p* < 0.001). The mediation effect was supported by a significant indirect effect (indirect effect B = 0.202, 95% CI [0.178, 0.230]), consistent with a statistical indirect association through depressive symptoms.

Figure 2c examines the influence of traditional bullying perpetration. The results indicated that bullying perpetration was positively associated with depressive symptoms (B = 0.157, *p* < 0.001), which in turn was related to suicidal behaviors (B = 0.853, *p* < 0.001). The direct effect of perpetration on suicidal behaviors remained significant (B = 0.193, *p* < 0.001), with an indirect effect of 0.134 (95% CI [0.113, 0.158]), consistent with a statistical indirect association through depressive symptoms.

Lastly, Figure 2d shows that cyberbullying perpetration was significantly associated with depressive symptoms (B = 0.124, *p* < 0.001), and depressive symptoms were again significantly related to suicidal behaviors (B = 0.864, *p* < 0.001). The direct effect between cyberbullying perpetration and suicidal behaviors was significant (B = 0.126, *p* < 0.001), with a corresponding indirect effect of 0.107 (95% CI [0.085, 0.132]), consistent with a statistical indirect association through depressive symptoms.

Together, these findings indicate that the associations between bullying involvement and suicide-related outcomes were consistent with statistical indirect associations through depressive symptoms across all four bullying subtypes.

### Tests of moderated mediation model

Figure 3 illustrates the results of four moderated mediation models testing whether parental supervision modulates the indirect pathway from school bullying (victimization and perpetration, traditional and cyber) to suicidal behaviors through depressive symptoms.

**Figure 3:**
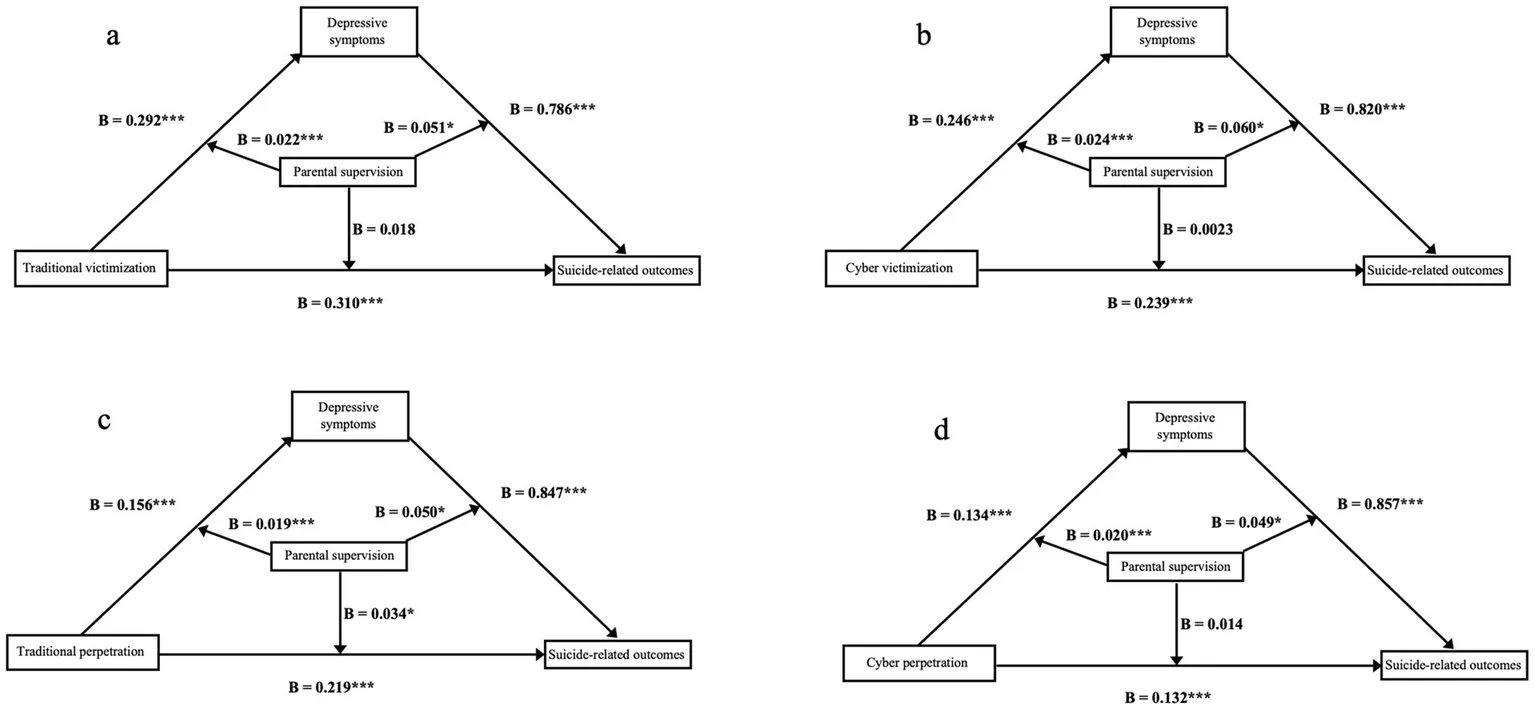
Path coefficients from moderated mediation models (PROCESS Model 59): **(a)** Traditional bullying victimization, **(b)** Cyberbullying victimization, **(c)** Traditional bullying perpetration, and **(d)** Cyberbullying perpetration. Perceived parental supervision was specified as a moderator of all three pathways. Dashed arrows indicate interaction terms, with gray dashed arrows representing non-significant interactions (*p* ≥ 0.05). Continuous predictors were standardized before analysis. Regression coefficients (B) are reported, with coefficients predicting suicide-related outcomes expressed in log-odds units. All models were adjusted for demographic and psychosocial covariates. ***p < 0.001, **p < 0.01, *p < 0.05.

In Figure 3a, which depicts the moderated mediation model for traditional bullying victimization, the interaction between traditional victimization and parental supervision in predicting depressive symptoms was statistically significant (B = 0.022, *p* < 0.001), indicating moderation in the first stage of the mediation process. Given that the interaction term was positive, this suggests that the positive association between bullying and depressive symptoms was stronger at higher levels of parental supervision. Additionally, parental supervision significantly interacted with depressive symptoms when predicting suicidal behaviors (B = 0.051, *p* = 0.039), suggesting second-stage moderation as well. However, the direct interaction between traditional victimization and parental supervision in predicting suicidal behaviors did not reach statistical significance (B = 0.018, *p* = 0.349).

In Figure 3b, which presents the moderated mediation model for cyberbullying victimization, parental supervision significantly moderated both stages of the mediation process. The interaction between cyberbullying victimization and parental supervision in predicting depressive symptoms was statistically significant (B = 0.024, *p* < 0.001), indicating first-stage moderation. Additionally, parental supervision significantly interacted with depressive symptoms in predicting suicidal behaviors (B = 0.060, *p* = 0.015), indicating second-stage moderation as well. The interaction between cyberbullying victimization and parental supervision in predicting suicidal behaviors was not significant (B = 0.002, *p* = 0.893).

In Figure 3c, parental supervision significantly moderated all three paths within the model of traditional bullying perpetration. First, the interaction between bullying perpetration and parental supervision was significant (B = 0.019, *p* < 0.001), indicating that the association between perpetration and depressive symptoms varied according to parental supervision. Second, the interaction between depressive symptoms and parental supervision was also significant (B = 0.050, *p* = 0.040), revealing moderation of the depression-to-suicidality pathway. Third, parental supervision moderated the direct association between perpetration and suicidal behaviors (B = 0.034, *p* = 0.016), with a stronger positive association observed at higher levels of parental supervision.

In Figure 3d, parental supervision significantly moderated two of the three paths in the model of cyberbullying perpetration. First, the interaction between perpetration and parental supervision was significant (B = 0.020, *p* < 0.001), indicating first-stage moderation from perpetration to depressive symptoms. Second, a significant interaction was also observed between depressive symptoms and parental supervision (B = 0.049, *p* = 0.042), reflecting second-stage moderation from depressive symptoms to suicidality. However, the interaction between cyberbullying perpetration and parental supervision in predicting suicidal behaviors did not reach statistical significance (B = 0.014, *p* = 0.301), suggesting no statistically significant moderation of the direct path.

Across all models, the point estimates of the conditional indirect associations through depressive symptoms increased from low to high levels of perceived parental supervision. For example, in the traditional victimization model, the estimated indirect association increased from 0.217 at low supervision to 0.254 at high supervision. A similar pattern was observed for the other bullying subtypes. However, these numerical differences do not necessarily indicate statistically significant differences between conditional indirect associations at different supervision levels. Furthermore, moderation of the direct association between bullying involvement and suicide-related outcomes was significant only for traditional bullying perpetration. Detailed conditional association estimates are presented in Table 2.

**Table 2:** Conditional direct and indirect associations between bullying involvement and suicide-related outcomes at low and high levels of perceived parental supervision.

Bullying type	Direct effect (Low / High)	Indirect effect (Low / High)
Traditional Victimization	0.303 [0.250, 0.356] / 0.323 [0.251, 0.395]	0.217 [0.195, 0.242] / 0.254 [0.223, 0.290]
Cyberbullying Victimization	0.238 [0.187, 0.290] / 0.241 [0.169, 0.313]	0.188 [0.164, 0.214] / 0.228 [0.194, 0.267]
Traditional Perpetration	0.205 [0.152, 0.257] / 0.243 [0.171, 0.315]	0.123 [0.102, 0.146] / 0.150 [0.119, 0.184]
Cyberbullying Perpetration	0.126 [0.071, 0.181] / 0.142 [0.066, 0.217]	0.105 [0.082, 0.132] / 0.133 [0.099, 0.173]

Values are presented as regression coefficients (B) with 95% confidence intervals (CI). All estimates are expressed on the log-odds scale. Indirect associations were estimated using PROCESS Model 59 with 5,000 percentile bootstrap resamples. Low and high parental supervision correspond to the 16th and 84th percentiles of the moderator, respectively. Confidence intervals for direct associations were obtained from the logistic regression models.

### Sensitivity analyses accounting for school clustering

An unconditional two-level random-intercept model yielded an intraclass correlation coefficient (ICC) of 0.064 for depressive symptoms, indicating that approximately 6.4% of the variance was attributable to differences between schools. In supplementary linear and logistic mixed-effects models accounting for school-level clustering, the directions and overall patterns of associations across the four bullying subtypes were broadly consistent with those observed in the primary PROCESS analyses. However, these sensitivity analyses did not provide school-adjusted bootstrap estimates of the indirect associations.

## Discussion

This cross-sectional study examined associations between school bullying experiences, depressive symptoms, parental supervision, and suicidal behaviors among Chinese adolescents. Our findings revealed that all forms of bullying involvement—both victimization and perpetration, in traditional and cyber contexts—were significantly associated with suicidal behaviors. Depressive symptoms were consistently associated with both bullying experiences and suicidal behaviors, supporting their potential mediating role in these relationships (Holt et al., 2015; Ye et al., 2023). Furthermore, the strength of these associations varied according to levels of parental supervision, with stronger associations observed among adolescents reporting higher supervision. These patterns suggest a complex interplay between peer victimization, family dynamics, and adolescent mental health that warrants further investigation through longitudinal and experimental research.

### The association between school bullying and suicidal behaviors

The findings of this study reinforce a robust association between school bullying and suicidal behaviors among adolescents, consistent with prior research on the psychological impacts of bullying (Bryson et al., 2021; Holt et al., 2015). Students who reported experiencing or engaging in school bullying—whether as victims or perpetrators, online or offline—also reported elevated rates of depressive symptoms and suicidal behaviors (Källmén and Hallgren, 2021; Stea et al., 2024). These patterns align with theoretical models suggesting that bullying may exacerbate feelings of isolation, rejection, and helplessness, which in turn may heighten vulnerability to self-harm or suicidal behaviors (Myklestad and Straiton, 2021).

It is important to note that the cross-sectional design of this study precludes causal conclusions about the direction of these relationships. Bullying experiences may contribute to the development of suicidal behaviors, but it is also possible that adolescents with pre-existing emotional vulnerabilities are more likely to become involved in bullying situations or to perceive peer interactions as threatening. Longitudinal research is needed to disentangle these temporal sequences and identify critical periods for intervention.

Nonetheless, these findings highlight the need for comprehensive school-based mental health services, peer support systems, and preventive anti-bullying strategies. Equipping students with emotional coping skills and fostering safe, inclusive school environments remain crucial steps toward reducing the co-occurrence of bullying experiences and suicidal behaviors.

### Depressive symptoms as a potential mediator

Our analyses revealed that depressive symptoms were significantly associated with both bullying experiences and suicidal behaviors, supporting their potential mediating role in these relationships. Adolescents who reported bullying involvement—whether as victims or perpetrators—also reported higher levels of depressive symptoms, which in turn were associated with greater likelihood of suicidal behaviors. These patterns were consistent across all four forms of bullying examined (traditional and cyber victimization and perpetration), suggesting a common psychological pathway that may link peer victimization to suicidal risk (Jiang et al., 2025; Klonsky et al., 2021).

Several psychological mechanisms may underlie these associations. First, bullying experiences may disrupt emotional regulation capacities, making it more difficult for adolescents to manage negative emotions such as anger, sadness, and fear (Vacca et al., 2023). This compromised regulatory capacity may contribute to the development of depressive symptoms. Second, bullying may trigger maladaptive cognitive processes such as rumination, where individuals repeatedly reflect on their negative experiences and perceived helplessness (Wu et al., 2026). This cyclical thought pattern may intensify depressive states and increase suicide risk.

However, it is crucial to emphasize that the cross-sectional nature of this study limits our ability to establish temporal precedence or rule out alternative causal pathways. The observed associations are consistent with a mediation model, but they do not confirm it. Depressive symptoms may precede bullying involvement, or both may be influenced by shared third variables such as family adversity, genetic predispositions, or early childhood trauma. Future longitudinal studies with multiple assessment waves are needed to test mediation hypotheses rigorously and to examine whether interventions targeting depressive symptoms can reduce the association between bullying and suicidality.

Despite these limitations, the strong associations observed in this study underscore the importance of early screening for depressive symptoms among adolescents exposed to bullying. School-based mental health programs should prioritize emotional resilience training, cognitive-behavioral interventions, and accessible counseling services to address the emotional consequences of peer victimization.

### Parental supervision and conditional associations

While parental supervision is often conceptualized as a protective factor, this study revealed a more complex pattern of associations. Higher levels of parental supervision were associated with stronger—rather than weaker—associations between bullying, depressive symptoms, and suicidal behaviors. Specifically, the association between depressive symptoms and suicidal behaviors was significantly stronger among adolescents who reported higher levels of parental supervision. Additionally, in most models, the association between bullying and depressive symptoms was also stronger under conditions of high supervision.

It is important to interpret these findings with caution. The cross-sectional design and the use of a brief two-item measure of parental supervision limit our ability to draw firm conclusions about the nature or direction of these associations. The parental supervision measure primarily captured adolescents’ perceptions of whether parents knew about their whereabouts and friendships, but it did not assess the quality, warmth, or autonomy-supportiveness of parental monitoring. Different dimensions of parenting may have divergent associations with adolescent mental health, and these dimensions were not distinguished in the current study.

One possible explanation for the observed pattern is that parental supervision in Chinese cultural contexts may be characterized by high involvement and close control, which—while intended as care—may be perceived by adolescents as pressure or intrusion, particularly in emotionally stressful situations (Huang and Wan, 2025; Sun et al., 2023). In some Chinese family contexts, parental involvement may be accompanied by an emphasis on authority and high expectations. Adolescents experiencing bullying may feel heightened fear of disappointing their parents or violating familial expectations, leading to intensified internalized distress and rumination rather than adaptive coping.

An alternative explanation is that higher levels of parental supervision may co-occur with greater emotional openness and parent–child communication, which could increase adolescents’ awareness and reporting of their own emotional distress (Hu and Zhou, 2025; Zimmer-Gembeck et al., 2014). Adolescents who feel closely connected to their parents may be more attuned to their internal states and more willing to acknowledge depressive symptoms and suicidal thoughts, whereas those with less supervision may underreport such experiences. In this interpretation, parental supervision does not amplify vulnerability per se, but rather increases the visibility of pre-existing distress.

A third possibility is reverse causation: parents may increase monitoring in response to signs of emotional distress or behavioral problems in their children. Adolescents who exhibit depressive symptoms or suicidal behaviors may elicit greater parental concern and supervision, creating an association that reflects parental response rather than a risk-amplifying effect of supervision itself.

Given these multiple interpretations and the limitations of the study design, we refrain from concluding that parental supervision causally increases vulnerability to bullying-related distress. Instead, we emphasize that the protective effects of parental monitoring are not uniform and may depend on the quality, style, and cultural context of supervision, as well as the adolescent’s developmental stage and emotional needs. Future research should employ longitudinal designs, multi-informant reports, and more nuanced measures of parenting dimensions to clarify these relationships.

### Implications for intervention

Despite the interpretive limitations inherent in cross-sectional data, these findings have several practical implications. First, they underscore the importance of addressing depressive symptoms as a key component of suicide prevention efforts in schools. Adolescents involved in bullying—whether as victims or perpetrators—should be routinely screened for depression and connected to appropriate mental health services.

Second, the findings suggest that family-based interventions should move beyond simply encouraging parental monitoring to fostering high-quality parent–child relationships characterized by warmth, autonomy support, and open communication. Parenting programs in Chinese contexts may benefit from incorporating culturally adapted strategies that balance parental involvement with respect for adolescent autonomy and emotional independence.

Third, interventions should adopt a systemic perspective that recognizes the interplay between individual, family, and school factors. Multi-level programs integrating anti-bullying policies, mental health literacy training for students and teachers, and family psychoeducation may be worth exploring in future intervention studies.

## Limitations

Several limitations should be noted when interpreting these findings. First and foremost, the cross-sectional design precludes causal inferences. The observed associations are consistent with mediation and moderation hypotheses, but they do not establish temporal precedence or rule out alternative explanations such as reverse causation or confounding by unmeasured variables. Longitudinal studies with multiple waves of data collection are needed to examine the temporal ordering of bullying, depression, parental supervision, and suicidality, and to test whether changes in one variable predict subsequent changes in others.

Second, the study relied exclusively on self-report measures, which are vulnerable to recall bias, social desirability bias, and shared method variance. Adolescents’ reports of parental supervision may reflect their subjective perceptions rather than objective parental behaviors, and their reports of depressive symptoms and suicidal behaviors may be influenced by current mood states. Future research should incorporate multi-informant data (e.g., parent and teacher reports) and objective or behavioral indicators of stress and suicidality to triangulate findings.

Third, parental supervision was assessed using a brief two-item scale that primarily captured parental knowledge of the adolescent’s activities and peer relationships. This measure does not distinguish between different dimensions of parenting, such as behavioral control, psychological control, warmth, and autonomy support, which may have distinct associations with adolescent mental health. More comprehensive assessments of parenting practices are needed to clarify which specific aspects of supervision are beneficial or potentially problematic.

Fourth, although supplementary multilevel analyses were conducted to account for school-level clustering, class-level identifiers were unavailable, and clustering within classes could not be explicitly modeled. Furthermore, school-adjusted bootstrap confidence intervals for the indirect associations were not estimated. Therefore, the robustness of the indirect associations to the hierarchical data structure remains uncertain. Future studies should account for clustering at both the school and class levels.

Fifth, the sample was drawn exclusively from Beijing, a large urban metropolis in China. The findings may not generalize to rural areas, other regions of China, or other cultural contexts where parenting norms, school environments, and mental health stigma may differ substantially.

Finally, the original anonymous survey did not include a dedicated protocol for identifying, referring, or following up with students who reported suicidal ideation, suicide planning, or suicide attempts. Although anonymity protected participants’ confidentiality, the absence of an individual-level risk management procedure limited opportunities to identify and provide timely support to students at potential risk. This represents an important ethical and procedural limitation. Future school-based studies assessing suicide-related outcomes should establish and document appropriate risk identification, referral, and follow-up procedures while safeguarding participants’ confidentiality.

## Conclusion

This cross-sectional study documented significant associations between school bullying, depressive symptoms, parental supervision, and suicidal behaviors among Chinese adolescents. Depressive symptoms were consistently associated with both bullying experiences and suicidality, and these associations were stronger among adolescents reporting higher levels of parental supervision. While these patterns are suggestive, the study design does not support causal conclusions. Longitudinal research with more comprehensive measures and multilevel analytic techniques is needed to clarify the temporal dynamics and causal pathways linking peer victimization, family relationships, and suicide risk. Nonetheless, these findings highlight the importance of integrated prevention efforts that address bullying, depression, and family dynamics in culturally informed ways to promote adolescent mental health.

## Data Availability

The datasets presented in this article are not readily available because the original datasets contain sensitive information about minors and mental health status, including suicidal behaviors. Due to ethical and legal restrictions on sharing such data, the raw datasets are not available for sharing. Aggregated, de-identified summary statistics and analysis code may be available from the corresponding author upon reasonable request and with appropriate institutional approval. Requests to access the datasets should be directed to xingyang@bjmu.edu.cn.
