# Supplementary material for: Associations between bullying, depressive symptoms, parental supervision, and suicidal behaviors in Chinese adolescents: a cross-sectional study
Source: Front Psychol. 2026 Oct 29;17:1984417. doi: 10.3389/fpsyg.2026.1984417 (PMC13754702; doi:10.3389/fpsyg.2026.1984417)
Supplement: Supplementary file 1 [file Table_1.docx]

Supplementary Table 1 Pearson Correlations Among Parental Supervision, Bullying Involvement, Depressive Symptoms, and Suicidal Behaviors

| **Variable** | **Parental supervision** | **Traditional victimization** | **Cyber victimization** | **Traditional perpetration** | **Cyber perpetration** | **Depressive symptoms** | **Suicidal behaviors** |
| --- | --- | --- | --- | --- | --- | --- | --- |
| **Parental supervision** | 1 |  |  |  |  |  |  |
| **Traditional victimization** | -0.167^***^ | 1 |  |  |  |  |  |
| **Cyber victimization** | -0.160^***^ | 0.636^***^ | 1 |  |  |  |  |
| **Traditional perpetration** | -0.161^***^ | 0.450^***^ | 0.428^***^ | 1 |  |  |  |
| **Cyber perpetration** | -0.139^***^ | 0.319^***^ | 0.453^***^ | 0.731^***^ | 1 |  |  |
| **Depressive symptoms** | -0.236^***^ | 0.325^***^ | 0.282^***^ | 0.179^***^ | 0.145^***^ | 1 |  |
| **Suicidal behaviors** | -0.124*** | 0.231*** | 0.213*** | 0.145*** | 0.117*** | 0.371*** | 1 |
| Note: Values represent Pearson correlation coefficients. ***p < 0.001. | | | | | | | |
